# 2025 European LeukemiaNet recommendations for the management of chronic myeloid leukemia

**DOI:** 10.1038/s41375-025-02664-w

**Published:** 2025-07-11

**Authors:** Jane F. Apperley, Dragana Milojkovic, Nicholas C. P. Cross, Henrik Hjorth-Hansen, Andreas Hochhaus, Hagop Kantarjian, Jeffrey H. Lipton, Hemant Malhotra, Dietger Niederwieser, Jerald Radich, Philippe Rousselot, Susanne Saussele, Charles A. Schiffer, Richard Silver, Simona Soverini, Leif Stenke, Anna Turkina, Luis Felipe Casado, Fausto Castagnetti, Francisco Cervantes, Richard E. Clark, Jorge Cortes, Michael Deininger, Timothy P. Hughes, Jeroen Janssen, Qian Jiang, Dong-Wook Kim, Richard A. Larson, Francois X. Mahon, Michael Mauro, Jiri Mayer, Franck E. Nicolini, Fabrizio Pane, Delphine Rea, Johan Richter, Gianantonio Rosti, Giuseppe Saglio, Rüdiger Hehlmann

**Affiliations:** 1 Centre for Haematology, Imperial College London, Hammersmith Hospital, London, UK; 2 Department of Haematology, Hammersmith Hospital, London, UK; 3 Human Development and Health, University of Southampton, Southampton, UK; 4 Department of Hematology, St Olavs Hospital, Department of Clinical and Molecular Medicine, Norwegian University of Science and Technology (NTNU), Trondheim, Norway; 5 Hematology/Oncology, Universitätsklinikum Jena, Jena, Germany; 6 Leukemia Department, University of Texas MD Anderson Cancer Center, Houston, TX USA; 7 Princess Margaret Cancer Centre, University of Toronto, Toronto, ON Canada; 8 Department of Medical Oncology, Sri Ram Cancer & Super-Specialty Center, Mahatma Gandhi Medical College Hospital, Mahatma Gandhi University of Medical Sciences & Technology, Jaipur, India; 9 Center for Cellular Immunotherapies, University of Pennsylvania & Penn Medicine, Philadelphia, PA USA; 10 Translational Science & Therapeutics Division, Fred Hutchinson Cancer Center, Seattle, WA USA; 11 Hematology Department, Centre Hospitalier de Versailles, Le Chesnay Rocquencourt, France; 12 III. Med Clinic, Medical Faculty Mannheim of the University of Heidelberg, Mannheim, Germany; 13 Karmanos Cancer Institute, Wayne State University School of Medicine, Detroit, MI USA; 14 Division of Hematology and Medical Oncology, Cornell Medicine, New York, NY USA; 15 Department of Medical and Surgical Sciences (DIMEC), Institute of Hematology Lorenzo e Ariosto Seràgnoli, University of Bologna, Bologna, Italy; 16 Department of Hematology, Karolinska University Hospital and Karolinska Institutet, Stockholm, Sweden; 17 National Medical Research Center for Hematology, Moscow, Russia; 18 Servicio de Hematología, HM Centro Integral Oncológico Clara Campal, Instituto de Investigación Sanitaria HM Hospitales, Madrid, Spain; 19 Institute of Hematology Seràgnoli, IRCCS Azienda Ospedaliero-Universitaria di Bologna, Department of Medical and Surgical Sciences, University of Bologna, Bologna, Italy; 20 IDIBAPS, Hospital Clínic, Barcelona, Spain; 21 Department of Molecular & Clinical Cancer Medicine, University of Liverpool, Liverpool, UK; 22 Georgia Cancer Center at Augusta University, Augusta, GA USA; 23 Department of Medicine, Division of Hematology/Oncology, University of Michigan, Ann Arbor, MI USA; 24 Precision Cancer Medicine Theme, South Australian Health & Medical Research Institute, Adelaide, and University of Adelaide, Adelaide, SA Australia; 25 Department of Hematology, Radboud University Medical Center, Nijmegen, The Netherlands; 26 Peking University People’s Hospital, Peking University Institute of Hematology, Beijing, China; 27 Department of Hematology, Eulji Medical Center, Eulji University, Daejeon, South Korea; 28 University of Chicago Comprehensive Cancer Center, Chicago, IL USA; 29 Département d’Hématologie, Institut Bergonié, University of Bordeaux, Inserm, UMR1312 Bordeaux, France; 30 Memorial Sloan Kettering Cancer Center, New York, NY USA; 31 University Hospital Brno and Masaryk University, Brno, Czech Republic; 32 Hematology Department and INSERM U1052, CRCL, Centre Léon Bérard, Lyon Cédex 08, France; 33 Department of Oncology and Hematology, University Federico II of Naples, Naples, Italy; 34 Département d’Hématologie, Hôpital Saint-Louis, APHP, Paris, France; 35 Department of Molecular Medicine and Gene Therapy, Lund University, Lund, Sweden; 36 IRCCS/IRST Dino Amadori, Meldola, FC Italy; 37 Department of Clinical and Biological Sciences, University of Turin, Turin, Italy; 38 Medizinische Fakultät Mannheim, Universität Heidelberg, Mannheim, and ELN Foundation, Weinheim, Germany

**Keywords:** Molecularly targeted therapy, Health care

## Abstract

In this 5th version of the European LeukemiaNet guidance for adult patients, there are important changes in several areas of management based on evidence available since 2020, including the World Health Organisation’s reclassification of CML as a biphasic disease. Previous advice to switch the tyrosine kinase inhibitor (TKI) on failure of molecular milestones, is modified to better account for individual patient circumstances. Our recommendations are summarized in tables designed to be read in conjunction with the text which offers justification and additional advice. We describe decision-making for first-line treatment, both in available drugs and their initial dosing. Similarly we elaborate on dose reduction rather than drug switching to manage toxicities and discuss treatment sequencing. Data have matured for the outcome of treatment discontinuation and for management of parenting for both men and women. We acknowledge that most patients will remain on treatment for many years and emphasize the needs to minimize side effects, manage co-morbidities and optimize quality of life. Recent advances in allogeneic stem cell transplantation have broadened access to alternative donors, and lessened limitations of age and co-morbidities such that transplant remains a valuable option for patients for whom long-term disease control is not achieved through TKI therapy.

## Introduction

The European LeukemiaNet (ELN) and other national and international groups have agreed on common definitions and standards for cooperative research, and guided management of patients with chronic myeloid leukemia (CML) worldwide for almost three decades [1–5] (NCCN Guidelines Version 3.2025: chronic myeloid leukemia). Hitherto the emphasis has been on preventing progression to the blast phase (BP) and prolonging survival, a strategy that has de facto led to some patients having sufficiently deep molecular responses to trial discontinuation of treatment. While maximizing the number of patients who may achieve treatment free remission (TFR) is a laudable goal, we also recognize that many patients will remain on life-long medication and for them, optimizing both their response and their quality of life is paramount. In this, the 5th iteration of the ELN recommendations for management of CML, we will highlight new information that directly affects patient care and move towards even more personalized treatment. Our recommendations are for adults only; guidelines for the management of pediatric patients have been published recently [6].

Our intention is not to repeat in detail previously available information and recommendations that are largely unchanged. Instead we will focus on the results and interpretation of new evidence-based data that are ready to be incorporated into patient care. References will be largely limited to those published since March 2020.

## Methods

Our consensus panel consists of 38 members from Europe, North America, Asia and Australia. The topics to be reviewed and potentially revised since our previous recommendations of 2020 [4] were identified through a systematic review of published literature from January 2019 to March 2025 using PubMed, Embase, Web of Science and the Cochrane Library, together with abstracts presented at the meetings of the American Society of Clinical Oncology (ASCO), the American Society of Hematology (ASH), the European Hematology Association (EHA) and the European School of Hematology—International CML Foundation (ESH-iCMLf). Search terms included chronic myeloid leukemia, tyrosine kinase inhibitors (TKI), the individual TKI, namely imatinib, dasatinib, nilotinib, bosutinib, ponatinib, asciminib, radotinib, and olverembatinib, treatment free remission (TFR) and allogeneic stem cell transplantation (alloSCT).

We followed an iterative process of distribution to panel members of key questions, collation of results and discussion of results via email and at 7 in-person and online meetings. We set a target of at least 75% concordance and recognize that this was not achieved in all instances. Remaining areas of controversy are discussed in the relevant sections. Funding for the meetings was provided by the ELN, a not-for-profit research network of excellence. We did not receive funding from any commercial source. Recommendations are restricted to TKI that have been approved for use in CML by national and/or international medicines agencies. We recognize that not all of these drugs are available worldwide due to absence of local approvals and/or prohibitive pricing.

## Recommendations

### Diagnosis

Our suggestions for the investigation of patients at diagnosis are largely unchanged and have been further clarified in the more detailed ELN manuscript on laboratory recommendations for the diagnosis and management of CML [7].

The hallmark of CML is the presence of the *BCR::ABL1* fusion gene, created by the t(9;22) which gives rise to the Philadelphia (Ph) chromosome abnormality, and diagnosis depends on its detection or inference, by cytogenetics (conventional chromosome banding analysis (CBA) or fluorescent in situ hybridization (FISH)) and/or by reverse transcriptase polymerase chain reaction (RT-PCR) assays. Table 1 describes the value and limitations of these methodologies. We continue to recommend morphology and CBA on bone marrow (BM) and RT-PCR on peripheral blood (PB) or BM at diagnosis, so as to accurately stage the disease, detect variant translocations, cryptic *BCR::ABL1* rearrangements and additional chromosomal abnormalities (ACA) and to precisely identify the *BCR::ABL1* transcript type to facilitate future monitoring of response to treatment [8]. Some laboratories use commercial kits for RT-PCR which do not recognize all rare transcripts: discrepant results such as negative RT-PCR with positive CBA should warrant further investigation. Additional detail can be found in Cross et al. [7].

**Table 1:** Recommendations for laboratory investigations at diagnosis.

Routine laboratory testing and physical examination	Full blood count, biochemistry including lipid profile, HbA1c, hepatitis B serology. ECG and assessment of spleen and liver size expressed by cm below the costal margin
Cytogenetics for t(9;22)(q34;q11) (85–95%) or variants (5–10%) involving one or both of 9q34 and 22q11 and other chromosomes	Reported in accordance with the International System for Human Cytogenetics Nomenclature. Bone marrow preferred, although karyotyping is possible on peripheral blood at diagnosis. Approximately 7% patients presenting in chronic phase (CP) have additional chromosomal abnormalities (ACA) which may have prognostic impact. ACA are more common at presentation in advanced phase.
Fluorescent in situ hybridization	Most useful as a diagnostic screening tool, usually performed on peripheral blood. Commercial probe sets detect *BCR::ABL1* fusions but do not identify ACA. Metaphase FISH is useful for detection of variant translocations and cryptic rearrangements.
Reverse transcriptase polymerase chain reaction assays: standard method of diagnosis	Required for the 1–5% with cryptic *BCR::ABL1* fusions not visible on conventional cytogenetic analysis but recommended for all patients Usually performed on peripheral blood. Methodology must be robust enough to detect all *BCR::ABL1* variants: discrepant cytogenetic and RT-PCR results require further investigation and referral to reference laboratories. Identification of variants at diagnosis essential for monitoring for residual disease. 98% of patients express e13a2 or e14a2 (e14a2 is the most frequent transcript). 10% of patients express both e13a2 and e14a2. e13a2 is more frequent in men and less frequent in older patients. 2% patients express alternative *BCR::ABL1* transcripts, the most common are e1a2, e6a2, e8a2, e19a2, e13a3, e14a3. Low levels of these variants in patients with predominant e13a2/e14a2 transcripts are most likely caused by alternative splicing and have no clinical significance.
Whole genome (WGS), whole exome (WES), targeted panels by next generation sequencing (NGS) for somatic mutations (reviewed in Ref. [9])	Somatically mutated genes are often found in CML (20-30%), more frequent in advanced phase than CP disease. Detection not routinely indicated at diagnosis in CP. Recommended in blast phase (BP) but somatic mutations are rarely actionable, exceptions may include *IDH1/2, NRAS, FLT3*. Mutations in genes associated with age-related clonal hemopoiesis can be acquired before or after emergence *BCR::ABL1* fusion gene. Can be performed on peripheral blood or bone marrow. Certain mutations are associated with either myeloid or lymphoid BP Some groups have reported an association of the presence of *ASXL1* mutations and poor outcome on TKI in CP.

*BCR::ABL1* point mutations associated with resistance (hereafter called *BCR::ABL1* mutations) are very rarely detected at diagnosis in chronic phase (CP) and testing is not recommended. Somatic mutations in genes other than *BCR::ABL1* are found in approximately 20–30% of patients, more commonly in advanced rather than chronic phase disease [9, 10]. To date the therapeutic implications of these mutations remain unclear and analysis should be regarded as a valuable research tool but not a diagnostic requirement in chronic phase. In contrast, and aligning with newly diagnosed acute leukemia, targeted panel next generation sequencing (NGS) is recommended for patients presenting in or progressing to BP.

### Disease classification

Traditionally CML is perceived as a triphasic disease, comprising chronic, accelerated and blast phases (CP, AP, BP). Attempts at more precise definitions of these disease stages have paradoxically led to disparities in the classification of the various phases. Since the introduction of the TKI, and the subsequent myriad of clinical studies, clinical colleagues largely adopted the long-standing definitions of BP (>30% blasts) of Karanas and Silver [11] and of AP by the MD Anderson Cancer Center (MDACC) [12], and incorporated these into clinical trial design and previous ELN recommendations. These are different from those in previous iterations of the World Health Organisation (WHO) Classification of Haematological Malignancies, and from the 2022 International Consensus Classification of myeloid neoplasms [13], predominantly in the percentage of blast cells in the bone marrow and/or peripheral blood used to distinguish the three phases and in the impact of ACA in Ph positive (Ph+) cells present at diagnosis or acquired during the disease course.

In 2022 the WHO revised the classification of disease phase in CML, omitting the concept of AP, and defining BP as the presence of >20% blasts [14]. Their rationale is persuasive. The distinction of the three phases by arbitrary cut-offs of blast percentages rather than by a better understanding of disease biology is less acceptable in the modern era of genomics. Multiple studies have shown that genetic changes in BP are rarely found in CP but are present in AP, suggesting that CML is a biphasic disease [15]. In practice most patients with either definition of AP at the time of diagnosis are treated with TKI and the majority have responses similar to those in CP, such that the classification does not change patient management. At least one study has shown that patients with blasts between 20% and 30% have an outcome more similar to those with blasts >30% than those between 10% and 20% and suggests that these patients may be candidates for more aggressive treatment including where possible, alloSCT [16]. The emergence of ACA in Ph+ cells in a patient with pre-existing disease is associated with an increased risk of disease progression [17], but the prognostic impact of ACA present at diagnosis in a patient with chronic phase disease is less clear and the type of the ACA may also be important [18–20]. Finally since the key diagnostic criterion for acute myeloid leukemia (AML) changed from ≥30% to ≥20% blasts in blood or marrow, progression to BP was no longer aligned with other chronic myeloid neoplasms. The WHO definitions recognize that patients can present with high- risk features detailed in Table 2, and that existing patients can develop changes predictive of impending disease progression that may impact treatment choices.

**Table 2:** Definitions of disease phase: ELN 2013, WHO 2016, ICC 2022, WHO 2022.

	Chronic phase (CP)	Accelerated phase (AP)	Blast phase (BP)
WHO 2016	Blasts (PB & BM) < 10%	Blasts (PB or BM) 10–19% PB basophils ≥20% Platelets <100 or >1000 × 10^9^/L unrelated or unresponsive to treatment Splenomegaly unresponsive to treatment Rising WCC unresponsive to treatment ACA at diagnosis including 3q26.2 rearrangements, +8, i(17q), +19, +Ph, complex karyotype ACA emerging on treatment	Blasts (PB or BM) ≥ 20% Extramedullary blast proliferation
ICC 2022 [12]	Blasts (PB & BM) < 10%	Blasts (PB or BM) 10–19% PB basophils ≥20% ACA at diagnosis or emerging on treatment including 3q26.2 rearrangements, +8, i(17q), +19, +Ph, complex karyotype	Blasts (PB or BM) ≥ 20% Myeloid sarcoma >5% lymphoblasts suggests lymphoid BP
WHO 2022 [13]	Blasts (PB & BM) < 20% **High risk indicators** **At diagnosis** High ELTS score Blasts (PB & BM) 10–19% PB basophils ≥20% ACA: 3q26.2 rearrangements, -7, i(17q) & complex karyotype Clusters of small megakaryocytes with fibrosis **High risk indicators** **On treatment** No CHR on 1st line TKI Resistance to 2GTKI (unless due to a *BCR::ABL1* mutation) Development of ACA Compound mutations in *BCR::ABL1*	No longer exists	Blasts (PB or BM) ≥ 20% Extramedullary blast proliferation Bona fide lymphoblasts in PB or BM (even if <10%)
ELN [3]	Blasts (PB & BM) < 15%	Blasts (PB or BM) 15-29% PB basophils ≥20% Platelets <100 × 10^9^/L unrelated to treatment Major route ACA emerging on treatment	Blasts (PB or BM) ≥ 30% Extramedullary blast proliferation

The proposed changes are controversial [21]. Counter-arguments include the long-standing acceptance and understanding of the phrase ‘acceleration’, which might indicate the need for a second generation TKI (2GTKI) from diagnosis, the inability to study AP patients in future clinical trials and difficulties in comparing outcomes of previous studies in all phases of the disease (which largely use the ELN criteria of previous recommendations) with those of future trials. The controversy is reflected in the opinions of our panel such that we cannot fully endorse either classification. However both acknowledge that there are patients who present with features of more advanced disease who may benefit from more potent drugs [22] and closer monitoring, and that evolution of disease in a patient is an indication of progression.

### Prognosis

Population based studies from Sweden and the Netherlands suggested that the life expectancy of patients with newly diagnosed CML now approaches that of the general population [23, 24]. However a more recent analysis of the Swedish CML registry has identified that small but significant losses in life-expectancy and quality-adjusted life expectancy remain across all age groups [25]. Identifying patients at higher risk of disease progression, providing vigilant response monitoring and managing adverse events are essential to optimize patient outcomes.

The previous risk scores, Sokal, Euro, and EUTOS, have been replaced by the EUTOS Long Term Survival (ELTS) score, which was developed to predict the probability of dying from CML in patients treated with a TKI, because most patients now die of unrelated causes [26–28]. A score calculator is accessible via https://www.leukemia-net.org/leukemias/cml/elts_score/

ACA are more frequent in BP and are referred to as major (+8, +Ph, i(17q), +19, +17, +21) or minor (−7/7q-, 11q23, 3q26.2) route abnormalities based on their frequency at the time of progression (> or <5% respectively). Together with complex aberrant karyotypes, they are considered high risk ACA and their prognostic power depends on the type of ACA, whether they occur alone or in combination and, possibly, on occurrence at diagnosis or later (see above). Some high-risk ACA are almost exclusively observed in combination such as +19 or -7/7q-; others emerge in the course of the disease and are rarely detected at diagnosis such as 3q26.2 abnormalities. We continue to consider the presence of high-risk ACA as a warning sign in chronic phase.

A possible impact of transcript type on response and survival has been analyzed extensively [7]. Studies suggesting higher rates of major molecular remission (MMR) and MR^4^ in patients with e14a2 compared to those with e13a2 have been shown to result, at least in part, from differing efficiencies in quantitative RT-PCR (RT-qPCR) amplification between the two transcripts [29, 30]. This can be corrected by using the droplet digital PCR (ddPCR) technique which is relatively insensitive to differences in amplification kinetics. It is less easy to explain the observations that patients with e14a2 are more likely to have successful trials of discontinuation than those with e13a2 transcripts [31, 32].

As discussed above somatic mutations in genes other than *BCR::ABL1* are not uncommon in CML, particularly in advanced phase disease [33]. *ASXL1* mutations are found in about 10% of patients and interestingly seem more frequent in children and young adults [10]. Some studies indicate that the presence of *ASXL1* mutations is associated with a poorer response to TKI and or reduced event-free survivals [13, 34–36] but as yet without impact on overall survival. *ASXL1* and other common somatic mutations in genes such as *DNMT3A* and *TET2* are also associated with age-related clonal hemopoiesis and their acquisition may antedate the emergence of *BCR::ABL1*; this can be confirmed by their continuing presence at the time of molecular remission.

### Monitoring response to treatment

Blood cell counts and differentials are required every 2 weeks until complete hematologic response (CHR) is achieved or more frequently in the event of hematologic toxicity. This applies not only to firstline treatment but also after switching TKI. Molecular monitoring to assess *BCR::ABL1* mRNA levels in blood cells is recommended at least every 3 months until major molecular remission is achieved and confirmed. Thereafter 4–6 monthly intervals between testing are justified if the patient remains in stable MMR or deeper response levels. More frequent molecular monitoring is advised if transcript levels fluctuate or rise, and when assessing eligibility for, and follow-up of, treatment discontinuation in selected patients.

Molecular response must be assessed according to the International Scale (BCR::ABL1^IS^) using *ABL1, BCR*, or *GUSB* as internal reference genes for patients expressing standard e13a2 and/or e14a2 *BCR::ABL1* mRNA variants (98% of CML patients) [7]. We maintain our previous definitions of disease response levels (Table 3). Deep molecular response (DMR) is defined as MR^4^ or deeper, irrespective of whether *BCR::ABL1* is detected or not. Tests should be optimized to enable the routine detection of MR^4.5^ in clinical samples.

**Table 3:** Definitions of response according to RT-qPCR or RT-ddPCR levels.

Terminology	BCR::ABL1^IS^	Minimum *ABL1* control transcript numbers	Minimum *GUSB* control transcript numbers
Equivalent to complete cytogenetic remission (CCyR)	≤1%	10,000	24,000
Major molecular response (MMR)	≤0.1%	10,000	24,000
4 log reduction from the IRIS standardized baseline (MR^4^)	≤0.01%	10,000	24,000
4.5 log reduction from the IRIS standardized baseline (MR^4.5^)	≤0.0032%	32,000	77,000
5 log reduction from the IRIS standardized baseline (MR^5^)	≤0.001%	100,000	240,000

Laboratory-developed tests with appropriate conversion factors, commercially available IS-calibrated kits and IS-calibrated whole systems such as the GeneXpert are all suitable in principle for molecular monitoring, provided they are validated locally for clinical use. RT-qPCR is the most commonly used method but RT-ddPCR is an acceptable alternative and may offer some technical advantages, particularly for assessment of very low-level disease.

Patients expressing atypical *BCR::ABL1* fusions (2% of CML patients) should ideally be monitored by personalized RT-qPCR and results expressed as individual molecular responses compared to baseline levels [37] or by RT-ddPCR assays [38].

In general CBA is insufficiently sensitive to monitor response but is recommended during follow-up of patients with rare transcripts that cannot be measured by RT-qPCR, at the time of evidence of resistance to TKI to exclude ACA and/or disease progression, and at progression to BP. FISH analyses can also be useful to monitor patients with rare or atypical transcripts.

### Milestones of response

The monitoring milestones of BCR::ABL1^IS^ at 3, 6, and 12 months specifically refer to the efficacy of the TKI and the advisability of switching treatment to achieve deeper responses: they do not address the need to change TKI because of the side effects of treatment. The milestones remain unchanged although our terminology has altered to emphasize their role in recognizing the risk of developing TKI resistance (Table 4).

**Table 4:** Response milestones for 1st, 2nd and 3rd line TKI expressed as BCR::ABL1^IS^.

	Favorable Low risk of developing resistance: treatment switch unnecessary	Warning Possible risk of developing resistance: treatment switch may become necessary	Unfavorable High risk of developing resistance: treatment switch preferred
Baseline	NA	High-risk ACA, high-risk ELTS score	NA
3 months	≤10%	>10%	>10% if confirmed within 1–3 months
6 months	≤1%	>1–10%	>10%-established resistance
12 months	≤0.1%	>0.1–1%	>1% (1–10%—see text for other considerations)
At any time	≤0.1%	>0.1–1% loss of ≤0.1% (MMR)	Loss of a previous response, resistant *BCR::ABL1* mutations, high-risk ACA

The phrases ‘optimal’, ‘warning’ and ‘failure’ are replaced by ‘favorable’ (treatment switch unnecessary), ‘warning’ (treatment switch may become necessary) and ‘unfavorable’ (treatment switch preferred). There are several reasons for this modification. First, no decision to switch treatment should be made on a single estimate of BCR::ABL1^IS^. All results should be interpreted in the context of previous results: for example, a treatment switch may be unnecessary in patients who have not reached a particular milestone at a pre-determined timepoint but whose successive results show a steady decline, so-called ‘late responders’. In contrast a rising RT-qPCR without an obvious explanation such as dose cessation/reduction, non-compliance etc should trigger CBA, kinase domain mutation analysis and a switch of TKI.

Second, the lack of a BCR::ABL1^IS^ <1% at 12 months has in previous recommendations been defined as ‘failure/resistance’ with the advice to switch treatment. Two recent studies have challenged this concept, particularly in older patients. In 131 patients with BCR::ABL1^IS^ > 0.1% after 2 years of TKI therapy, patients with levels >0.1%–1% or >1%–10%, had 10-year CML-specific survival rates of >90%. Patients with levels >10% had a worse but not very poor outcome with a 10-year survival rate of 80% [39]. Prompted by this observation, landmark survival analyses were performed for 1342 evaluable patients in the German CML IV study, who achieved BCR::ABL1^IS^ < 0.1%, 0.1%–1%, >1%–10% or >10% at 3, 6, 12, and 24 months. Ten-year survivals of patients who would have been termed “treatment failure” because of BCR::ABL1^IS^ >10% at 3 and 6 months and/or >1% at 12 months were approximately 80%, some 10% less than patients with optimal responses. In contrast in patients in whom the RT-qPCR was >10% at 12 months the OS dropped to 55%. Older patients (defined as >60 years) understandably had poorer survival than younger patients but were dying largely of non-CML related diseases [40]. These findings need confirmation in other settings but might suggest a more conservative approach in those with co-morbidities (usually the more elderly), whose BCR::ABL1^IS^ are in the unfavorable categories, but in whom a switch to a drug with more troublesome side effects may cause harm. We are reluctant to recommend different approaches simply because of patient age as management should be individualized and many patients >60 years will be in good health and without co-morbidities. For these and younger patients who can tolerate more potent drugs without toxicity, and who may be pursuing treatment free remission, a *BCR::ABL1* mutation analysis and a switch of TKI remain our recommended approach.

A change of TKI at 3 months due to *BCR::ABL1*^IS^ >10% (lack of early molecular response (EMR)), if confirmed by a subsequent sample at least 4 weeks later, has been controversial, largely because there has been no evidence for a better outcome following a TKI switch. In the aforementioned landmark analysis of >800 patients within the German CML IV study with BCR::ABL1^IS^ results available at 3, 6, 12 and/or 24 months, 223 (28%) and 104 (12%) failed to achieve BCR::ABL1^IS^ <10% at 3 and 6 months respectively, but remained on imatinib: the majority achieved this milestone at a later date [40, 41]. In the French SPIRIT study, long-term survival of approximately 80% was observed in patients with BCR::ABL1^IS^ > 10% at 3 months [42]. However, the 5-year outcome of the DASCERN study in which patients without EMR were randomized to remain on imatinib or change to dasatinib shows that patients randomized to dasatinib were more likely to achieve MMR and MR^4^ (77% and 53% respectively) than patients who remained on imatinib (44% and 31% respectively) although both overall and progression-free survivals were excellent in both groups (>95%). Patients in the imatinib arm were allowed to cross over to dasatinib at the point of subsequent failure of treatment, and although 65% obtained MMR only 4% achieved MR^4^ [43]. We have maintained the lack of confirmed EMR at 3 months in the unfavorable category but emphasize the importance of addressing the caveats mentioned above (kinetics of RT-qPCR responses, co-morbidities) plus any dose modifications in those 3 months and/or lack of compliance, before switching the TKI.

Finally, thought should be given to the motivation for changing treatment, which is sometimes related to the desire to achieve DMR and offer the patient a trial of TKI discontinuation. We have known for more than a decade and confirmed recently in a large Italian population based study, that achieving the molecular milestones in the first 12 months predicts for deep molecular responses at a later date [44]. Patients who do not achieve these milestones are less likely to have a successful TFR. A recent review of the German population registry showed that TFR was attempted in 24%, 23%, and 26% of patients who satisfied the 3, 6, and 12 month milestones respectively, compared to only 3–6% of those who did not achieve the milestones [45]. This would suggest that most, but not all, patients destined for an attempt at TFR can be identified in the first 12 months. Striving for a TFR that is highly unlikely by introducing more potent but also more toxic drugs may not be in the patients’ best interests.

Additional molecular monitoring may be indicated if the kinetics of the response are not clear, or if toxicity or intolerance cause dose interruptions or reductions. The same definitions are recommended for second-line and third-line treatment. A persistent suboptimal treatment response, including “warning”, to one or more TKIs is an indication for mutational screening (see below) and the need to address patient compliance.

### Resistance and *BCR::ABL1* mutations

An unfavorable response to TKI therapy occurs in approximately 15–20% of patients treated in first line, and in up to 50% of patients in later lines. Multiple factors, not necessarily mutually exclusive, can contribute to TKI resistance [46]. In some patients however, less than favorable responses may be related to poor compliance with treatment and this should always be considered whenever response is unsatisfactory. Therapeutic drug monitoring, when available, may help to evaluate patient exposure to a given drug and/or identify drug–drug interaction.

*BCR::ABL1* mutation testing is indicated in the case of TKI resistance or early signs that resistance may be developing (warning in Table 4), progression to or presentation in, BP and in the case of relapse after alloSCT if a mutation was detectable prior to transplant. In contrast, recurrence after TFR has almost never been associated with selection of mutations and *BCR::ABL1* mutation testing is not recommended in patients who lose MMR after a TFR attempt.

*BCR::ABL1* mutations are, at present, the only actionable mechanism of resistance. Positivity for a mutations should usually trigger a change of therapy, and detection of specific mutants helps exclude TKI that are unlikely to be effective (Table 5). A number of studies have reported relative sensitivities to various TKI and provide potentially valuable information: none are comprehensive and all are generated via in vitro models that may not always be reproduced clinically. *BCR::ABL1* mutations that line or map close to the myristoyl-binding pocket, including A337V/T, L340Q, A344P, A433D, G463D/S, P465S/Q, V468F, F497L, I502L/N, and V506L/M specifically confer resistance to asciminib, but should be sensitive to ATP-competitive TKI [47–50]. Interestingly some mutations mapping farther away, in the ATP-binding pocket or N-lobe, such as M244V, Q252H, and F359V, also confer resistance to asciminib. although the mechanisms have not been fully elucidated. In case of the M244V; the mutation appears to stabilize the active conformation of the kinase, thereby interfering with the action of asciminib [51, 52]. Furthermore the functional integrity of both the SH3 and SH2 domains of ABL1 are required for BCR::ABL1 inhibition by asciminib. Because *ABL1* exon 2 (a2) encodes 23 amino acids of the SH3 domain, patients with rare transcripts that do not contain the *ABL1* exon 2 (most commonly e13a3 and e14a3) are expected to be resistant to asciminib [53, 54].

**Table 5:** Recommended tyrosine kinase inhibitors in case of *BCR::ABL1* mutations.

M244V	Nilotinib, dasatinib, bosutinib, ponatinib
Y253H	Dasatinib, bosutinib, ponatinib, asciminib
E255K/V	Dasatinib, ponatinib, asciminib
V299L	Nilotinib, ponatinib, asciminib
T315I	Ponatinib, asciminib
F317L/V/I/C, T315A	Nilotinib, bosutinib, ponatinib, asciminib
F359V/I/C	Dasatinib, ponatinib
A337V/T, L340Q, A344P, A433D, G463D/S, P465S/Q, V468F, F497L, I502L/N, V506L/M	Any ATP-competitive TKI

Resistance can be due to the development of compound mutations, i.e., two mutations in *cis* in the same *BCR::ABL1* molecule. Based on in vitro IC_50_-based predictions and on preliminary in vivo evidence, it appears that the great majority of compound mutations will be resistant to imatinib and to second-generation TKI, with the highest level of resistance in those that include T315I. Moreover, some T315I-inclusive compound mutations have been reported in both ponatinib- and asciminib-resistant patients [55, 56].

Sanger sequencing has long been the gold standard for *BCR::ABL1* mutation analysis although the sensitivity is acknowledged to be low, with mutated clones forming <20% of the total leukemic burden being unlikely to be detected. Targeted NGS provides a more accurate assessment of mutation status, and because it can be employed at BCR::ABL1^IS^ > 0.1%, it allows early detection of emerging mutations below the threshold for detection by Sanger sequencing [57]. Moreover, when chemistries generating relatively long reads are used, NGS theoretically enables clonal analysis, hence straightforward identification of compound mutations. We recommend cDNA-based NGS testing for *BCR::ABL1* mutation screening, provided that a reliable and validated assay has been established (although commercial myeloid gene panels frequently include *ABL1*, their use is not recommended). Given that implementation of routine NGS-based *BCR::ABL1* mutation testing may be challenging, the use of Sanger sequencing is acceptable whenever NGS is not available or accessible and does not represent inappropriate patient management. Sensitive ddPCR tests may also be implemented for defined actionable mutations [58].

Detailed laboratory recommendations addressing the technical aspects of *BCR::ABL1* mutation testing more extensively have been provided in the ELN publication of Cross et al. [7].

### First-line treatment

Six TKI are currently approved for first-line therapy, the first generation TKI imatinib, three 2GTKI, dasatinib, nilotinib, and bosutinib, the 4th generation drug, asciminib, in some countries and radotinib in South Korea.

Multiple randomized trials (16 or more) have now compared frontline therapy with imatinib versus 2GTKI [4]. The rates of CCyR, MMR, DMR and transformation to AP or BP all favor 2GTKI but none of the studies showed an OS benefit [59, 60]. This may be because of the availability of effective subsequent-line therapies that rescue the patients and rebalance their outcome favorably. The more rapid achievement of DMR may lead to earlier attempts at TFR but it is as yet unclear whether shorter durations of treatment will result in the same level of success. This is being tested in several on-going studies. A recent Japanese prospective randomized study compared nilotinib and dasatinib in 454 newly diagnosed patients. They found no statistically significant differences in the rates of EMR, CCyR, MMR, DMR, discontinuation rates or PFS and OS suggesting that both drugs are equally effective when used in firstline [61].

Unlike ATP-competitive TKI, asciminib binds to the ABL1 myristoyl pocket. In the ASC4FIRST study, 405 newly diagnosed patients were randomized to asciminib 80 mg daily or a TKI of investigators choice. At a median follow-up of approximately 15–16 months, the rates of MMR at weeks 48 and 96 were superior with asciminib (67.7%/74.1%) versus investigator selected TKI (49%/52%) and also in the subgroups of asciminib (69.3%/76.2%) versus imatinib (40.2%/47.1%), and asciminib (66%/72%) versus 2GTKI (57.8%/56.9%). Grade ≥3 adverse events and those leading to discontinuation by 96 weeks were lower with asciminib (44.5%, 5%) than with imatinib (49.5%, 13.1%) and 2GTKI (59.8%, 12.7%) [50, 62]. The rates of arterial occlusive events (AOE) were 0% in the imatinib arm and 2–3% in asciminib and 2GTKI treated patients. Long-term survival rates are not yet available but these data have resulted in FDA approval for first line therapy in the USA in 2024. Availability and affordability is preventing the uptake of first-line asciminib elsewhere but where these are no obstacles asciminib presents a further useful choice in first-line therapy.

Prior to the introduction of the TKI, interferon-alfa (IFN) was the standard of care for patients ineligible for alloSCT. It provided good count control in the majority and induced CCyR in a minority of, usually younger, individuals. Several studies explored the combination of imatinib or a 2GTKI and IFN in first-line treatment and showed either no or a modest improvement in the achievement of molecular endpoints, and no differences in OS [42, 63]. Improvements were offset by an increase in toxicity often necessitating IFN dose reduction or cessation, such that the addition of IFN cannot be routinely recommended. This is supported by the results of the phase III TIGER study, which is an initial randomization between nilotinib or nilotinib plus pegylated IFN, followed by discontinuation of nilotinib in patients in the combination arm who achieve sustained MMR at 2 years. The MMR rates at 2 years again showed a modest improvement for the combination arm at 93% compared to 89% for nilotinib alone but any improvement in MMR rates using IFN was offset by impaired tolerability [64].

The TKI labels recommend starting doses of imatinib of 400 mg daily, dasatinib 100 mg daily, bosutinib 400 mg daily, and nilotinib 300 mg twice daily. These regimens were derived from the traditional development of cancer drugs, which identifies the maximum tolerated dose (MTD) in phase1/early phase 2 studies and conducts the phase 2 and pivotal trials at one dose level below the MTD. More recently some investigators have explored starting the TKI at lower than standard doses Compared with the historical data using dasatinib 100 mg daily, dasatinib 50 mg daily resulted in similar or perhaps better rates of responses and reduced the rates of pleural effusions and myelosuppression [65]. This particular dosing regimen has not yet been tested in a prospective randomized study and cannot be formally recommended. In a prospective study of reducing the dasatinib dose to 50 mg daily in patients with high drug plasma levels, showed similar efficacy and a reduced incidence of pleural effusions compared to patients therapeutic levels who remained on 100 mg daily [66]. The 2-year follow-up of the DASAHIT study in which patients commencing dasatinib were randomized to 100 mg daily or 100 mg daily on 5 days of each week, showed that patients treated in first-line had similar rates of MMR and cumulative toxicity but pleural and pericardial effusions occurred less frequently in the experimental arm [67]. A study from Japan evaluating dasatinib 20 mg daily in patients 70 years and older showed good early efficacy but long-term follow-up is not yet available [68]. Starting bosutinib at 400 mg daily results in a high rate of gastro-intestinal side-effects (early self-resolving diarrhea in the first 2–4 weeks) and early discontinuation. Commencing treatment at a lower dose may reduce the rate of treatment discontinuation and improve compliance. A suggested dose-adjusted bosutinib schedule might comprise 100–200 mg daily for 1–2 weeks, 300 mg daily for 1–2 months, increasing to 400 mg daily as indicated by the side effects and treatment response [69].

The choice of the TKI in the frontline therapy of CML should consider several factors. The first is the aim of therapy, overall survival or treatment free remission (TFR). For some older patients OS may be the important aim, and TFR, if achieved over time would be a welcome event. In younger patients a lifetime of therapy may be a concern, and TFR may be as important as OS as a treatment endpoint. Quality of life data generated in the EuroSKI study also showed that younger patients (defined as age <60 years at time of stopping) benefited more from treatment discontinuation than older patients [70]. Thus an argument can be made for starting a 2GTKI as first-line therapy in younger individuals. Secondly, patients with high ELTS scores may benefit from a more potent TKI upfront. Thirdly, some co-morbidities might be exacerbated by specific TKI. Patients with pulmonary disease should avoid dasatinib. Patients with gastro-intestinal problems or with renal or liver dysfunction may chose to avoid bosutinib. Nilotinib is inadvisable in patients with diabetes mellitus, high or very high cardiovascular risk including a history of vaso-occlusive events (VOEs) or pancreatitis. Although some studies have identified less favorable outcome with certain *BCR::ABL1* transcript types, e.g. e1a2, others report contrasting results, and transcript type should not influence therapeutic decisions [71, 72]. Finally in many countries where patients are wholly or partly self-funding, consideration of cost is important although the availability of generics has lessened this concern.

### Second-line treatment

Several studies and ‘real world’ data show that approximately 30–40% of patients change their first-line TKI because of intolerance or resistance. Since the previous version of these recommendations there have been several publications discussing the benefits and safety of managing adverse events in responding patients (at least MMR) by dose reduction. Imatinib can be reduced from 400 mg to a range of 100–300 mg daily; dasatinib from 100 mg daily to a range of 20–50 mg daily and nilotinib from 300 mg twice daily to 150–200 mg twice daily or even 150–200 mg once daily [73–76]. After dose reduction, patients should be monitored closely to be certain that the level of response is maintained. There are however particular side effects which are serious and prohibitive and require a change of TKI therapy rather than dose reduction. These include recurrent pleural effusions despite dose reductions, pulmonary hypertension, venous or arterial occlusive events (VOE or AOE), enterocolitis, serious neurologic conditions (e.g. dementia, Parkinsonism) and immune-mediated myocarditis, hepatitis or nephritis.

After first-line imatinib and in the absence of specific *BCR::ABL1* mutations any suitable 2GTKI can be used as they appear equally effective in second-line, although no studies have directly compared the 2GTKI with each other. The choice is driven by factors such as age, lifestyle, comorbidities and potential future adverse events. The presence of specific *BCR::ABL1* mutations are the main driver for the selection of a TKI active against that mutation (Table 5). The choice and dose of the possible TKI are discussed in more detail in the next section.

After resistance to a first-line 2GTKI and in the absence of specific *BCR::ABL1* mutations, the use of an alternative 2GTKI is rarely successful in achieving molecular responses [49, 77, 78] and consideration should be given to early use of ponatinib or asciminib (although the latter is not currently licensed for second-line therapy in all countries).

Responses (milestones) to second-line treatment are the same as to first-line treatment.

### Treatment beyond second-line

For patients who are intolerant to first- and second-line therapy even after dose reduction, it is reasonable to try an alternative 2GTKI, if necessary introducing it at a lower dose and titrating up depending on tolerability and response.

For patients resistant to their second line drug, asciminib or ponatinib should be the first choice, co-morbidities and *BCR::ABL1* mutations permitting. Both have been trialed in single arm Phase I/II studies and showed valuable response rates. Results from the ponatinib phase II PACE study were discussed in previous versions of these recommendations with additional information about dosage provided by the recently completed OPTIC trial. Patients with BCR::ABL1^IS^ > 1% were randomized one of three doses of ponatinib, 45 mg, 30 mg or 15 mg, with mandatory dose reduction to 15 mg in the 45 mg and 30 mg arms once transcript levels dropped below 1%^IS^. CCyR rates at 12 months were 44.1%, 29.0% and 23.1% in the 45 mg, 30 mg and 15 mg cohorts respectively. Grade 3 or greater arterial occlusive events were less common than in the original phase II study of ponatinib (at 4.3%, 4.3% and 3.2% in each of the 45 mg, 30 mg and 15 mg arms respectively), which may reflect patient selection and/or improved management of cardiovascular co-morbidities. Sub-group analyses suggest that where possible, for patients with a *BCR::ABL1* mutation, particularly T315I, or for those with BCR::ABL1^IS^ >10%, the starting dose should be 45 mg [79].

In the Phase I dose-finding study of asciminib in 115 patients who had failed at least two TKI (intolerance and/or resistance) and were without the T315I mutation, the majority received a starting dose ≥40 mg daily and some as high as 200 mg twice daily. At a median follow-up of 5.9 years, 70 (60%) patients remained on treatment and of patients evaluable for achievement of MMR, 65% achieved at least that level of response. Asciminib was generally well tolerated with 13% discontinuing for adverse events. The most frequent side effects ≥grade 3 were increased lipase (21.7%), arterial hypertension (18.3%), and thrombocytopenia (10.4%) [80]. A further report described 45 evaluable patients with T315I mutations who received higher doses of asciminib (150–200 mg bd): 62% achieved or maintained BCR::ABL1^IS^ > 1% including 48% and 81% of ponatinib-pretreated and -naive patients respectively [81]. Early results from dose-finding studies of asciminib in combination with one of either imatinib, dasatinib or nilotinib demonstrated early efficacy but with increased side effects [82].

The superiority of asciminib over a 2nd or subsequent 2GTKI was clearly demonstrated in ASCEMBL, a randomized study of bosutinib vs. asciminib in patients who had failed (intolerance and/or resistance) at least 2 prior TKI. The rates of CCyR and MMR following asciminib compared to bosutinib at 96 weeks were 39.8% vs. 16.1%, and 37.6 vs. 15.8% respectively; and adverse events were fewer after asciminib treatment [49]. The value of switching to asciminib in cases of ponatinib resistance and vice versa is less clear and eligible patients should be referred for consideration of alloSCT at the time of resistance to either [83, 84].

Olverembatinib, a new ATP-binding site competitor, designed to have specific activity against T315I, is approved for use in 3rd and subsequent line treatment and/or for patients with T315I mutations in China [85]. In the Phase I/II studies in 127 patients in CP, the cumulative incidences of CCyR, MMR, MR^4^, and MR^4.5^ at 3 years were 69%, 56%, 44%, and 39% respectively. The highest response rates were observed in patients with a single T315I mutation. Side effects included hyperpigmentation, hypertriglyceridemia, proteinuria, and thrombocytopenia. The incidences of cardiovascular events, overall and grade 3/4 were 32% and 11.5%, which will require further scrutiny in on-going phase III studies [86]. These encouraging results were confirmed in a Phase Ib single arm study of olverembatinib in CP patients resistant to at least two TKI, with MMR rates in patients with prior ponatinib and asciminib resistance of 43% and 33% respectively [87].

The definition of an acceptable response to third and subsequent line treatment is arguable, although a BCR::ABL1^IS^ > 1% has long been considered insufficient for optimal survival. The increasing use of first-line 2GTKI and treatment switches for intolerance have confounded this issue. For patients treated initially with imatinib and who work through 2nd and even subsequent generation TKI for intolerance, or for patients who have demonstrated resistance but for whom the goals of treatment are the achievement of responses optimizing survival, the milestones for response, at least to third-line TKI, should be identical to those for first- and second-line treatment. In patients in chronic phase who are eligible for alloSCT, donor searches should be commenced at the time of resistance to a 2GTKI and referral for transplant made at the time of resistance to ponatinib and/or asciminib.

### Advanced phase disease

Presentation in advanced phase is rare in World Bank high and upper-middle income countries but common in lower-middle and low income nations, probably reflecting delayed diagnosis. Whether ELN or WHO classifications of disease phase are adopted, both recognize indicators of high-risk progression in both newly presenting and existing CP patients (Table 2). Although data are limited, it seems that patients presenting in advanced phase have a more favorable prognosis compared to patients who progress from chronic phase on TKI therapy. Flow cytometry to determine the BP phenotype may inform treatment as lymphoblastic transformation has more treatment options and a better outcome, although attention to any possible central nervous system involvement is required. Treatment for eligible patients in BP is by intensive combination chemotherapy with a TKI, ideally dasatinib or ponatinib [88, 89], followed by alloSCT. Patients who are not candidates for intensive chemotherapy combinations may benefit from low intensity chemotherapy combined with TKI and several such approaches are now in clinical trial.

### Allogeneic stem cell transplantation

The use of alloSCT in CML decreased dramatically following the introduction of TKI, but it remains an important therapeutic modality for patients in CP resistant to third and/or fourth generation TKI, or intolerant to all available TKI and for those who present in or progress to, BP.

The outcome of alloSCT has improved considerably during the TKI era and now older patients and/or those with co-morbidities can be transplanted using reduced intensity or non-myeloablative conditioning. The use of haploidentical related donors has increased the donor pool such that almost every patient will have a donor. In addition, risk factors for alloSCT such as disease duration, recipient age and donor type described in the pre-TKI era may no longer be valid. In a recent analysis of 904 patients, overall and progression-free survivals were influenced only by disease stage (CP1 vs. >CP1, HR 1.5), Karnofsky performance score (KPS) (>80 versus ≤80%, HR 0.5), but not by the number of lines of TKI [90].

Wherever possible transplant should be performed in first CP as outcomes following alloSCT are considerable inferior for patients in advanced phases. We recommend consideration of transplant in CP1, which initially involves identification of a suitable donor, most usually through the local transplant center, at the time of resistance to the first 2GTKI, whether this be given first- or second-line. This is particularly important in the presence of single or compound mutations resistant to multiple TKI or the emergence of ACA as these may be harbingers of disease progression. The transplant may subsequently be deemed unnecessary if the patient responds to the next TKI but there can be a considerable delay in identifying a suitable donor, so it is best to be prepared.

For patients presenting in or progressing to BP, long-term outcome with any of the currently available TKI is poor, as is the outcome of alloSCT in BP [91]. Every possible effort should be made to regain a second chronic phase and offer alloSCT promptly thereafter [88]. Orti et al. recently reported hazard ratios (HR—reference of 1 for CP1) for overall survival for unrelated donor transplants of 2.25, 1.63 and 1.58 for BP, AP and >CP1 respectively [92]. Transplant of resistant BP patients, unless on study, is not recommended. The risk of relapse after alloSCT for CML rises with increased immunosuppression (required for higher levels of HLA disparity) and reduced intensity conditioning regimens. Where possible we recommend myeloablative conditioning.

There are several unanswered questions concerning management after alloSCT, not least of which is the definition of molecular relapse. Following alloSCT we recommend molecular monitoring at least 3 monthly in the first years post-transplant. The frequency can be reduced to 6 monthly if the patient has molecularly undetectable disease but should continue lifelong. The use of a TKI prophylactically after alloSCT for high risk CP1 is arguable, as residual resistant disease in theory should remain resistant. The availability of newer TKI with more limited resistance profiles can be considered for patients with residual or emerging positivity for *BCR::ABL1* transcripts. If unavailable or unsuccessful in restoring molecularly undetectable disease, escalating dose donor lymphocyte infusions can be effective. In patients transplanted in advanced phase, continuation of the TKI given before transplant to restore a chronic phase is advised when engraftment is secure. More difficult is the decision to discontinue post-transplant TKI but after a prolonged period, say 2 years, of sustained negativity stopping is a not unreasonable approach.

### Treatment free remission

Stopping treatment for an attempt at TFR is usually a safe procedure at centers with access to high-quality molecular monitoring and with careful patient selection [93]. Some patients otherwise eligible for TFR prefer to remain on therapy, and it is important that clinicians discuss the available data with patients before a TFR attempt begins.

Multiple trials of TKI discontinuation have been conducted, each with slightly different entry criteria and different triggers for restarting the TKI, but with very similar results: approximately 40–50% of patients can remain off treatment [94]. Consistent inclusion criteria in most studies were a minimum duration of TKI therapy of 3 years and a sustained DMR of at least 1 year. Recently, the EuroSKI study reported the final analysis of 728 patients: MMR rates at 6 and 12 months were 61% and 46% respectively [95]. The DESTINY study, in which patients reduced their TKI dose to 50% of the standard dose for 12 months before stopping reported a TFR rate of 72% [96]. One advantage of this approach is that it may, in the case of loss of MMR, allow reinstitution of TKI-therapy at the reduced dose given immediately prior to stopping. To date most patients attempting TFR and reported in the literature, were treated with firstline imatinib. After stopping nilotinib or dasatinib as either first or second-line therapy, the probability of maintaining TFR has also been approximately 50% [97–99]

In highly motivated patients with a high priority for TFR who have not yet achieved DMR, such as younger patients or women who wish to become pregnant, a change to a more potent TKI is reasonable, although there are no data to suggest that such a strategy improves the success of TFR.

Loss of MMR has been the trigger for restarting therapy in most studies [100] and for more than 80% this will occur in the first 6–8 months after stopping, emphasizing the need for frequent monitoring and structured follow-up during this early period (Table 6). Confirmation of loss of MMR on a second occasion is not usually necessary and could delay restarting therapy. Some patients have fluctuating values between MMR and MR^4^ which sometimes improve over time without restarting TKI and such patients require careful serial monitoring. About 90–95% of patients who experience molecular recurrence regain their previous DMR after restarting TKI therapy. Usually, the same TKI is restarted, unless prior side-effects indicate a reason for change. Few of the many thousands of patients in TFR trials have had poor outcomes: 6 cases of sudden BP in the TFR setting were reported from France. The risk of BP in this situation was estimated at ≤0.1% [101].

**Table 6:** Guidance for attempts at treatment discontinuation.

Requirements for tyrosine kinase inhibitor discontinuation in CP CML.	
Mandatory:	CML in first CP only (data are lacking outside this setting).
	Motivated patient with structured communication.
	Access to high quality molecular monitoring using the International Scale (IS) with rapid turn-around of results. In case of atypical transcripts in laboratories with a high standard of quantification.
	Patient’s agreement to more frequent monitoring after stopping treatment.
Minimal (stop allowed):	First-line therapy, second-line if the reasons for switch were intolerance or resistance due to a mutation sensitive to another TKI.
	Typical e13a2 or e14a2 *BCR::ABL1* transcripts. In case of atypical transcripts in laboratories with a high standard of quantification.
	Duration of TKI therapy >5 years (>4 years for 2GTKI).
	Duration of DMR (MR^4^ or better) >2 years.
Optimal (stop recommended for consideration):	Duration of TKI therapy >5 years.
	Duration of DMR >3 years if MR^4^.
	Duration of DMR >2 years if MR^4.5^.
Procedures after stop:	Molecular monitoring 6 to 8 weekly for the first 6 months, 2 monthly for months 6–12, and every 3–6 months thereafter. Monitoring should increase in frequency if there is an increase in *BCR::ABL1* transcript levels.
	Restart TKI-therapy if MMR is lost.
	If TKI-therapy is restarted monitor 4-6 weekly until MMR is regained and then every 3 months until MR^4^ is regained.

Late loss of MMR has been reported in up to 14% of patients more than 2 years after stopping [102, 103] such that long-term monitoring is recommended. Of note are the different kinetics of early (rapid) relapses versus late (slow) recurrences [104].

A number of parameters have been reported to influence TFR success. The final analysis of Euro-SKI confirmed durations of TKI treatment and DMR before TKI stop as significant factors for predicting MMR loss at 6 months. In addition, expression of the e14a2 transcript was identified as a good prognostic factor for maintaining TFR. For late MMR losses after 6 months, TKI treatment duration, percentage of blasts in peripheral blood and platelet count at diagnosis were significant factors in multivariate analysis. For the entire study period of 36 months, multiple logistic regression models identified duration of treatment, percentage of blasts at diagnosis, and transcript type as independent factors for MMR maintenance. Other groups have shown that the depth of remission at the time of stopping TKI, particularly if the evaluation was by the more sensitive ddPCR, can also predict success [105, 106] The mechanisms which prevent recurrence are poorly understood: current studies focus on possible immune-mediated control of residual disease.

Stopping TKI-therapy in patients who failed their first attempt is possible although limited data are available to help to decide on the duration of re-treatment prior to the second stop. In general the TFR rates in reported studies were lower than in studies of first TFR attempt, but still at a level which is clinically meaningful. In contrast the interim analysis of the DASTOP study described 62 patients who experienced molecular recurrence after their first attempt at TFR: they were re-treated for 3 years, at least 2 of which were with dasatinib and the probabilities of TFR at 6, 12 and 24 months were 61%, 56% and 46%, respectively [107]. Early recommendations advised against stopping in patients who had demonstrated prior resistance, usually to imatinib, but this can also be successful, albeit at a lower rate than patients without resistance. Similarly two recent reports confirm successful TFR in selected patients with ACA at diagnosis [108, 109]. Stopping treatment for patients expressing atypical *BCR::ABL1* fusions may be considered if sensitive, quality-controlled molecular monitoring available, e.g. persistent molecularly undetectable disease for >2 years and ≥4 log reduction from baseline levels.

A ‘withdrawal syndrome’ comprising polymyalgia and/or arthralgia occurs in 20–30% of patients on TKI cessation. This is usually mild and self-limiting but can necessitate treatment with nonsteroidal anti-inflammatory drugs and on occasions, short courses of corticosteroids. Rarely the symptoms do not settle until the reintroduction of the TKI.

Our recommendations for TKI discontinuation are summarized in Table 6: these differ from previous guidance in the frequency of monitoring after treatment discontinuation.

### Parenting

The switch in treatment goals from the prevention of disease progression to optimization of quality of life has in turn resulted in a focus on safe parenting for both men and women. Although data have accumulated since the last version of these guidelines, we acknowledge that parenting events while on TKI remain infrequent and our discussions with our patients should reflect the limitations of our knowledge.

**Male patients:** there is no consistent or convincing evidence from animal models or the clinical experience to date of any impact of TKI on male fertility nor on the outcome of the pregnancies in female partners or on the development of their offspring. A small number of human studies reported reduced sperm counts and in one, an increased frequency of abnormal sperm morphology in men after several months exposure to a TKI. However these studies lacked paired samples taken prior to starting a TKI so causality was not established. A small study of paired samples before treatment and after a median of 61 months from 11 patients did not show any impairment in sperm concentration or motility [110]. A systematic literature review of 428 pregnancies in the partners of 374 men who parented children while taking imatinib or 2GTKI, reported 400 live births (93.5%) with a congenital abnormality rate of 2.5%, similar to that seen in the general population [111]. Recent data from the Incyte Biosciences pharmacovigilance database also report the absence of congenital abnormalities in children conceived when their fathers were taking ponatinib [112].

We recommend that men taking TKI should continue treatment when attempting conception.

**Female patients:** the teratogenicity of imatinib was reported in 2008 and since then women with established CML with unplanned pregnancies were advised to discontinue their TKI at the time of the first positive pregnancy test while those planning pregnancy were advised to discontinue prior to attempting conception. Since these recommendations the incidence of reported congenital abnormalities has declined but this more likely reflects altered awareness of the risks, and changes in management, by both patients and physicians, rather than any reduced risk from later generation TKI. The risk of fetal damage is assumed to begin at the time of implantation, about 15 days after fertilization and to continue until completion of organogenesis (around gestational week 16). Imatinib and nilotinib cross the placenta at concentrations considerably lower than that achieved in the mother whereas the concentration of dasatinib in fetal blood was 75% of maternal levels in the one case studied [113].

Congenital abnormalities similar to those originally reported with imatinib have been described for dasatinib and nilotinib: information is limited for bosutinib and asciminib. Two cases of Hirschsprung’s disease, one with additional unspecified renal insufficiency have recently been reported in women taking ponatinib at the time of conception, despite the ponatinib being discontinued at 7 and 9 weeks gestation [112]. The manufacturers of all the available drugs used in treating CML recommend that TKI should be discontinued during pregnancy.

Rarely women with CML present in pregnancy. Chelysheva et al. recently reported 87 patients presenting in pregnancy: normal childbirth occurred in 66 women (76%) with an incidence of congenital malformations no higher than in the general population. Treatment was given in 43 of the 66 pregnancies, predominantly interferon-alpha (often from diagnosis to delivery) and imatinib (always after the 16th gestational week) without deleterious effects. Women were less likely to elect for abortion and more likely to receive active treatment in the years 2012–2022 compared to the previous decade, reflecting our increasing confidence in managing pregnancy in CML [114]. Data from Robertson et al. reported that intervention by leucapheresis, IFN or both, was more likely in women presenting with high white cell counts (WCC) leading to the suggestion that patients with WCC < 100 × 10^9^/L can be initially managed by a period of watchful waiting to assess the count dynamics before introducing therapy [115]. For most patients with low or intermediate ELTS scores CML is unlikely to progress over the remaining period of the pregnancy. In both studies most women responded well to the introduction of TKI during or after pregnancy.

We continue to recommend that, in general, TKI should not be used during pregnancy. IFN, including pegylated forms, can be used safely during pregnancy and can be given for count control, recognizing that IFN is slow acting. The ability of IFN to provide any degree of molecular remission in less clear, certainly in newly diagnosed patients and even maintenance of previous MMR cannot be assumed. Hydroxycarbamide is known to be teratogenic in animals but evidence for this in humans is lacking [116]. It is infrequently given during pregnancy, except over short periods to reduce counts rapidly: international guidelines recommend against its use in pregnant women with sickle cell disease [117].

However, we also acknowledge that there are situations in which lack of effective disease control is potentially harmful to both mother and fetus and where treatment decisions are more nuanced. In the past we have recommended stopping the TKI prior to conception, but given our awareness of the effects on organogenesis it is also reasonable for women in CP with regular menstrual cycles and access to pregnancy tests, to continue their TKI until the first positive pregnancy test, approximately 4 weeks from their last menstrual period and defined as 4 weeks of gestation. There are a number of case reports in addition to the ELN series, in which imatinib and nilotinib have been introduced/re-introduced after gestational week 16, without apparent harm. Dasatinib should be avoided throughout pregnancy and data are too limited for the remaining drugs to support their use. TKI should not be used during breast feeding so the duration of this should be adapted according to disease response (and the need to start a TKI) at delivery.

Women in advanced phase who are of child-bearing age will be candidates for high dose chemotherapy and alloSCT. Delay to treatment will adversely affect their chances of survival and in this situation termination of the pregnancy should be discussed, and the wishes and decision of the patient and her family respected.

Women with CML who contemplate or become pregnant while on TKI, are heterogeneous in terms of age, disease phase, reproductive history, financial and social circumstances and current response to treatment but also in their ability to access licensed TKI and accurate and regular molecular monitoring. The advice given to any woman must take account of her personal situation and wishes. Access to and collaboration with, informed obstetric advice is essential.

Table 7 provides a rationale for different management strategies for women in chronic phase.

**Table 7:** Guidance for management of pregnancy in women with CML.

a: Planning a pregnancy in established CML	
Current response	Recommendation
≥MR^4^ (RT-qPCR ≤ 0.01% IS)	Manage as for TFR • Remains in TFR—leave off treatment indefinitely irrespective of pregnancy. • Becomes pregnant and loses MMR (Table 7b). • Does not become pregnant and loses MMR—restart same or more potent drug after discussing possible adverse events and regain original deep remission. Further attempts at conception possible at a later date.
MMR but not MR^4^ (RT-qPCR > 0.01% IS < 0.1% IS).	Continue TKI to achieve ≥MR^4^ and manage as above. If MMR is established with sufficient follow-up to suggest achievement of MR^4^ is unlikely, then there are 3 possible scenarios. 1 Discontinue TKI and manage any subsequent pregnancy (Table 7b). 2 Continue TKI and obtain regular pregnancy tests, Discontinue TKI at first positive test and manage as in Table 7b. 1 Continue TKI with patient stopping after completion of menses & taking a pregnancy test 2 weeks later. If positive stay off TKI, manage as in Table 7b. • Second and third options only possible if the patient understands the risks, and access to regular molecular monitoring and pregnancy tests.
<MMR (RT-qPCR ≥ 0.1% IS)	• Continue on the same or a more potent TKI to establish a deeper response before attempting conception. • In older patients consider referral to a local IVF service. TKI can be interrupted to enable a hyperstimulation cycle. Embryos can be implanted fresh or frozen. If implanted immediately, manage as in Table 7b. If frozen, try to establish a deeper response before implantation and manage as for ≥MMR. • If the patient wishes to pursue pregnancy when not in MMR: manage as in second and third scenarios detailed above for MMR.

b: Managing the pregnancy in established CML Discontinue the TKI at confirmation of pregnancy. Refer to obstetrics and explain need for early and regular fetal scanning RT-qPCR & full blood count every 6–8 weeks, adjust as clinically indicated		
Current response	Recommendation	
	Weeks 0–16	≥Week 16
MR^4^: RT-qPCR ≤ 0.01% IS MMR: RT-qPCR ≤ 0.1% IS MR^2^: RT-qPCR ≤ 1% IS	• Continue observation without therapy. • IFN can be introduced at any point to control counts. Ability to maintain molecular responses is unproven. • If RT-qPCR is increasing rapidly and/or loss of CHR and after week 16, start imatinib 400 mg daily. Nilotinib up to 400 mg daily can be used in case of imatinib resistance or intolerance.	
No MR^2^: RT-qPCR > 1%	Commence IFN	If loss of CHR Imatinib 400 mg daily Nilotinib up to 400 mg daily if imatinib resistant/intolerant

c: Presentation of CML chronic phase in pregnancy Assess risk by ELTS score and the presence/absence of additional chromosomal abnormalities.	
Asymptomatic with WCC < 100 ×10^9^/L, low or intermediate ELTS and absence of ACA	• Suggest period of watchful waiting to determine kinetics of disease. If slowly evolving it is reasonable to avoid treatment, especially in the 1st trimester. • Please note WCC < 100 ×10^9^/L is an arbitrary recommendation and management depends on trajectory of counts & symptoms. • IFN can be introduced at any point.
Symptomatic and/or WCC < 100 ×10^9^/L, high ELTS, additional ACA present	Up to at least week 16 the patient can be managed by • Leucapheresis alone—will depend on service availability and intravenous access. • IFN alone. • Leucapheresis in combination with IFN. After week 16 • Introduce imatinib 400 mg daily or nilotinib if imatinib intolerant • Consider acetylsalicylic acid and/or low molecular weight heparin for patients with thrombocytosis

### Adverse events

The vast majority of patients, even those destined for a successful attempt at TFR, will take TKI for many years. With more TKI available and improved knowledge regarding potential side effects, much of patient management is focused on preventing and/or minimizing the toxicities and optimizing quality of life.

Adverse events, as opposed to direct drug toxicity, may also occur as a result of pre-existing co-morbidities, interactions with other medications, or simply a result of the aging process. It is beyond the scope of this publication to describe in detail all known side-effects of the individual TKI (Table 8) and the potential impact of other disease processes and their treatment. Members of this panel have contributed to extensive reviews of these issues, together with recommendations for baseline and periodic monitoring of co-morbidities and we will not attempt to repeat their advice [118, 119].

**Table 8:** Noteworthy side effects with relative incidence and severity.

	IM	DAS	NIL	BOS	PON	ASC	OLV
Hematological side-effects	+	++	(+)	+	++	(+)	++
**Biochemical abnormalities**							
ALT	+	0	+	++	+	0	+
Bilirubin	0	0	+^a^	0	0	+^a^	(+)
Creatinine	+	0	0	+	0	0	(+)
Cholesterol	0	0	++	0	0	0	0
Glucose	0	0	+	0	0	0	++
Low thyroxine (T4)	0	0	0	0	+	0	?
**Vascular side-effects**							
Arterial thrombotic events	0	(+)	+	0	++	0	++
Arterial hypertension	0	(+)	+	0	++	(+)	++
Pulmonary arterial hypertension	0	+	0	(+)	0	0	(+)
**Miscellaneous side-effects**							
Dermatitis	+	+	++	+	+	(+)	++
Skin pigmentation	+	0	0	0	0	0	+++^b^
Pleural effusion^c^	(+)	++	(+)	+	(+)	(+)	(+)
Pericardial effusion	0	+	0	+	0	0	?
Periorbital edema	++	0	0	0	0	0	(+)
Fluid retention	+	0	0	0	0	0	(+)
Diarrhea	+	(+)	0	+++^d^	0	0	(+)
Pancreatitis	0	0	+	0	(+)	(+)	0
Fatigue	+	+	+	+	+	+	+
Muscle cramps	++	0	0	0	0	0	0
**Some newly recognized rare side-effects**							
GAVE^e^	(+)	0	0	0	0	0	0
Follicular lymphoid hyperplasia	0	(+)	0	0	0	0	0

This is a non-extensive list of clinically relevant side effects occurring in different incidence and severity between TKIs used for treatment of CML. Many more, like headache, nausea, constipation and myalgia, occur at similar rates and frequently are self-limited. The relative differences are indicated by + signs. This is based on expert opinion of mainly first line studies. Please see [118, 119] for details on side effect management and occurrence. Unexpected AEs may not be caused by TKIs and should be investigated as clinically indicated.

*IM* imatinib, *DAS* dasatinib, *NIL* nilotinib, *BOS* bosutinib, *PON* ponatinib, *OLV* olverembatinib, *ALT* Alanine aminotransferase. 0 very rare or not described, (+) rare, + not infrequent ++ frequent, +++ very frequent and ? no available data.

^a^Unconjugated, harmless, common in Gilbert’s syndrome.

^b^Pigmentation change, more common in non-white populations.

^c^Can occur late.

^d^Often short term.

^e^GAVE: gastric antral vascular ectasia, an uncommon cause of an upper GI bleed.

Initial reporting of drug toxicities emerges from clinical trial data. Because of the relatively limited follow-up of most of these studies they may not capture more delayed events. Drugs initially deemed to be “safe” or with very low incidences of some side effects, can later produce higher rates of certain toxicities, some of which can impair both quality and duration of life.

Before commencing treatment, irrespective of the line of therapy, patient assessment should include a detailed medical and drug history together with relevant examination and investigations. Our recommendations for tests before and during therapy are given in Table 9. Co-morbidities should be identified and adequately treated. The chosen TKI should be both effective and safe for each individual patient. We discussed earlier the potential to reduce drug dosage in patients who have toxicities but who are otherwise responding well. In contrast, should their disease fail to respond well or indeed progress, and mandate the introduction of more potent and potentially more toxic drugs, this should be discussed with the patient.

**Table 9:** Recommendations for investigations before and during TKI therapy.

a: Routine additional baseline investigations prior to TKI therapy: minimum requirements							
Cardiovascular							
	**Imatinib**	**Dasatinib**	**Nilotinib**	**Bosutinib**	**Ponatinib**	**Asciminib**	**Olverembatinib**
ECG^a^	√	√	√	√	√	√	√
Lipid profile, HbA1C	–	–	√	–	√	–	√
BP	√	√	√	√	√	√	√
TTE	–	–	–	–	–	–	–
**Other**							
HBV^b^ (HBsAg, HBcAb)	√	√	√	√	√	√	√
TFT	–	–	–	–	√	–	√
Lipase +/- Amylase	As clinically indicated						
**b: Additional monitoring investigations on TKI therapy: minimum requirements**							
**Cardiovascular**							
	Imatinib	Dasatinib	Nilotinib	Bosutinib	Ponatinib	Asciminib	Olverembatinib
ECG	As clinically indicated						
Lipid profile, HbA1C	–	–	6–12 monthly	–	6–12 monthly	–	6–12 monthly
BP	√	√	√	√	√	√	√
TTE	As clinically indicated						
**Other**							
TFT	–	–	–	–	6–12 monthly		6–12 monthly
Lipase +/- amylase	As clinically indicated						

*ECG* electrocardiogram, *BP* blood pressure, *TTE* transthoracic echo, *TFT* thyroid function test.

^a, b^These tests do not need to be repeated upon starting an alternative TKI, unless clinically indicated. Further screening tests such as TTE, ankle-brachial index (ABI), Duplex ultrasound scan (DUS) can be performed according to physician’s discretion and clinical need. BP monitoring is recommended in general as good clinical practice.

The process of monitoring for toxicity, identifying new medical conditions and their treatment is as important as monitoring disease response. Patients may not always inform their CML healthcare professionals of other medical problems, and vice versa, fail to tell other specialists about their CML and its treatment. Prescriptions may be dispensed at different pharmacies so that the back-up of the well-informed pharmacist may be lost.

In summary we recommend a comprehensive approach to the health of our patients with referral to other specialties as appropriate. We have recognized some important toxicities only after several years of the use of some of the TKI. There should be a ‘best’ drug for most patients: one size does not ‘fit-all’ and carefully considered choices and actions will yield the best results for patients.

## Summary

Twenty-five years have elapsed since imatinib became widely available to the CML community, over which time we have observed a very remarkable change in patient prognosis, only possible through the work of earlier researchers in the fields of genetics, cytogenetics, biochemistry and molecular biology alongside the efforts of the clinical observationists, triallists and transplant pioneers. We now have several highly effective oral agents with at least three more entering clinical trials this coming year. The additional choice has made excellent clinical outcomes possible for the majority of patients with access to multiple agents but more options can make management decisions more challenging. Since the last version of these recommendations, drug choices are wider and we now have a better understanding, on one hand of the potential for treatment discontinuation and on the other, the impact of co-morbidities and the need to optimize quality of life. In this manuscript we have provided the evidence while emphasizing the need to put the patient at the heart of decision- making. We have made very considerable progress but significant challenges remain, not least of which is the prevention and management of blast phase disease.

However, for much of the world’s population, treatment choices are not only dependent on evidence but also on drug availability and cost. In most countries there is today a striking price difference among various *BCR::ABL1* TKI. Generic imatinib is usually by far the most cost-effective alternative, the annual price for 400 mg daily being as low as $200 to $500, potentially less than annual monitoring costs. Currently generic dasatinib is more expensive with annual costs varying between $3000 and $100,000. Patented second and third generation TKI are considerably more expensive, with approved first-line drugs at annual price ranges of $20,000 to $300,000, with still no demonstrable survival benefit between imatinib and other TKI. Some second, third and fourth generation drugs may facilitate earlier TFR, be superior for high-risk CML and/or be better tolerated. Whether these suggestions can be confirmed and translated into a clear gain in quality-adjusted life years (QALY) compared to imatinib within acceptable cost frames requires further investigation. Until that time recommendations such as these provide a framework for management and an evidence-based common-sense approach to patient care.
